# The Cardiovascular System in Space: Focus on In Vivo and In Vitro Studies

**DOI:** 10.3390/biomedicines10010059

**Published:** 2021-12-28

**Authors:** Ronni Baran, Shannon Marchal, Sebastian Garcia Campos, Emil Rehnberg, Kevin Tabury, Bjorn Baselet, Markus Wehland, Daniela Grimm, Sarah Baatout

**Affiliations:** 1 Department of Biomedicine, Aarhus University, Ole Worms Allé 4, 8000 Aarhus, Denmark; 201709730@post.au.dk (R.B.); daniela.grimm@med.ovgu.de (D.G.); 2 Department of Astronomy, Catholic University of Leuven, 3000 Leuven, Belgium; shannon.marchal@student.kuleuven.be; 3 Radiobiology Unit, Belgian Nuclear Research Centre (SCK CEN), Boeretang 200, 2400 Mol, Belgium; emil.rehnberg@sckcen.be (E.R.); kevin.tabury@sckcen.be (K.T.); bjorn.baselet@sckcen.be (B.B.); 4 Department of Microgravity and Translational Regenerative Medicine, Otto von Guericke University, Universitätsplatz 2, 39106 Magdeburg, Germany; s.garcia@campus.tu-berlin.de (S.G.C.); markus.wehland@med.ovgu.de (M.W.); 5 Research Group ‘Magdeburger Arbeitsgemeinschaft für Forschung unter Raumfahrt- und Schwerelosigkeitsbedingungen’ (MARS), Otto von Guericke University, Universitätsplatz 2, 39106 Magdeburg, Germany; 6 Department of Molecular Biotechnology, Ghent University, 9000 Ghent, Belgium; 7 Department of Biomedical Engineering, University of South Carolina, Columbia, SC 29208, USA

**Keywords:** cardiovascular disease, microgravity, cosmic radiation, spaceflight, simulated microgravity

## Abstract

On Earth, humans are subjected to a gravitational force that has been an important determinant in human evolution and function. During spaceflight, astronauts are subjected to several hazards including a prolonged state of microgravity that induces a myriad of physiological adaptations leading to orthostatic intolerance. This review summarises all known cardiovascular diseases related to human spaceflight and focusses on the cardiovascular changes related to human spaceflight (in vivo) as well as cellular and molecular changes (in vitro). Upon entering microgravity, cephalad fluid shift occurs and increases the stroke volume (35–46%) and cardiac output (18–41%). Despite this increase, astronauts enter a state of hypovolemia (10–15% decrease in blood volume). The absence of orthostatic pressure and a decrease in arterial pressures reduces the workload of the heart and is believed to be the underlying mechanism for the development of cardiac atrophy in space. Cellular and molecular changes include altered cell shape and endothelial dysfunction through suppressed cellular proliferation as well as increased cell apoptosis and oxidative stress. Human spaceflight is associated with several cardiovascular risk factors. Through the use of microgravity platforms, multiple physiological changes can be studied and stimulate the development of appropriate tools and countermeasures for future human spaceflight missions in low Earth orbit and beyond.

## 1. Introduction

On Earth, humans are subjected to a constant gravitational force that has been an important determinant of human evolution and function. During spaceflight, astronauts are subjected to a prolonged state of microgravity (µ*g*) that induces a myriad of physiological adaptations such as bone loss, muscle atrophy, fluid shifts, and decreased plasma volume as well as cardiovascular deconditioning, leading to orthostatic intolerance (OI). OI has been identified as one of the main postflight problems in astronauts, with an incidence of approximately 80% after long-duration spaceflight missions [1]. In addition, a compromised cardiovascular system is more susceptible to other space hazards and therefore more prone to pathophysiological changes [2]. Considering the harmful effects of the µ*g* environment on human organ systems (e.g., the cardiovascular and musculoskeletal systems) and support systems (e.g., life support systems), it is necessary to develop appropriate tools and countermeasures for successful space missions [3].

The International Space Station (ISS) provides researchers with the best, well-rounded platform to study the effects of the space environment on the human body. However, not all experiments can be done in space due to restraints in time, costs, equipment, and manpower. Therefore, space analogues play a crucial role in complementing our understanding of the space environment and to test countermeasures for near-term spaceflight research. A space analogue is a situation on Earth that induces physical, biological, mental, and emotional effects on the body that are similar to those experienced in space [3]. They allow researchers to study the effects of µ*g* on human benign cardiovascular cells, stem cells, and other specialized cells such as among others cancer cells, without the use of human subjects [4,5].

This review aimed to summarise current research and knowledge in the field of space life science with a focus on the cardiovascular system in the µ*g* environment. The results from space-based research platforms have been supplemented with ground-based research results, which is divided into in vivo experiments (e.g., head-down bed rest studies) and in vitro experiments (e.g., random positioning machine [RPM]).

## 2. Spaceflight Hazards 

### 2.1. Isolation and Confinement

The space environment induces a host of physiological, biomedical, and environmental stressors on crew living and working in space. Space psychology is an emerging discipline that aims to support crew health and well-being through the application of psychological and behavioural countermeasures. Psychological countermeasures include astronaut selection, training, and in-flight support. Multiple human elements have been identified that can potentially threaten crew productivity, health, and mission success. These include various psychological stressors (1), psychosocial stressors (2), human factors (3), and stressors related to the habitability of the spacecraft (4) [6].

1. Isolation and confinement are examples of psychological stressors. They induce a variety of psychological and physical effects, such as motivational decline, fatigue, somatic complaints (i.e., insomnia and headaches), and social tensions [6]. Two studies have correlated long-term isolation and confinement with strained crew relations, heightened friction, and social conflicts [7,8]. Further psychological stressors are the limited possibilities for rescue, high-risk conditions, and sleep disruptions [9].
2. Multicultural and multinational factors are seen as important psychosocial stressors that have been related to preflight, inflight, and postflight incidences. National cultures influence several aspects of crew performance, such as communication, technology interfacing, religion and holidays, habitat aesthetics and work, management, and leadership style [10]. In addition, psychosocial stressors that have been identified are interpersonal tensions between crew and/or ground stations, disruptions in family life, and crew factors such as age, personality, etc.
3. Space human factors involve the study of the interface between humans and technology. This discipline applies the principles by which humans interact with intelligent machines/tools. Several principles to take into account include hand–eye coordination, cognition, information processing, and memory capabilities. Other human factors are high/low levels of workload, danger, and risks associated with equipment failure or malfunctions [9].
4. The habitability of the spacecraft refers to a multitude of areas, including architecture, clothing, crew displays/interfaces, housekeeping, hygiene, lighting, and privacy, among others [11]. The integration and support of human, technological, environmental, and mission elements promote crew performance, safety, and physical and psychological health during long-duration spaceflight. Other stressors related to the habitability are chronic exposure to vibrations/noise, limited sleep facilities, and isolation from support systems [6].

### 2.2. Space Radiation

The radiation environment in and around the ISS is formed by three main sources of radiation: galactic cosmic rays (GCR), solar cosmic rays, and particles from the Van Allen radiation belts [7]. The amount of ionising radiation depends on several factors, such as altitude, the solar cycle, and shielding of the modules of the ISS [8].

The ISS is in a low Earth orbit (LEO) at an altitude of approximately 400 km and with an orbital inclination of 51.6°. Higher altitudes are less protected by the geomagnetic field against charged particles [8]. The radiation belts are formed by charged particles captured by the geomagnetic field and form two diffuse bands. The inner belt contains high concentrations of electrons (hundreds of kiloelectron volts) and energetic protons (hundreds of megaelectron volts (MeV)) and extends from an altitude of 1600 to 13,000 km (0.2–2 Earth radius (RE)). The outer belt contains mainly high-energy electrons (0.1–10 MeV) and extends from an altitude of 19,000 to 40,000 km (3–10 RE). The ISS is protected by Earth’s magnetic field and is located below the inner radiation belt. Radiation fluxes increase substantially as the altitude of the ISS increases. The orbital inclination means that the station passes through the South Atlantic Anomaly (SAA) every day. The Earth’s geomagnetic field at the SAA is weaker, resulting in energetic particles penetrating to lower altitudes. When the ISS passes through the SAA, the astronauts are exposed to higher levels of ionising radiation [8,10].

The ISS radiation environment changes according to changes in solar activity. Solar flares and coronal mass ejections produce high fluxes of charged particles, which are brought to Earth by solar wind and are mostly deflected by Earth’s magnetic field. These solar particle events and the timing of them impact the radiation environment and may significantly increase the radiation dose for the crews in LEO. During solar maximum, the increased solar activity can compress Earth’s magnetic field and push the magnetic field lines and the Van Allen radiation belts to lower altitudes. During solar minimum, the decrease in solar activity will attenuate the interplanetary magnetic field generated by the sun and thus lead to higher fluxes of GCR to hit Earth’s magnetic field [10,11].

Astronauts receive approximately 80 millisievert (mSv) of radiation during a 6-month period to the ISS, whereas people on Earth receive approximately 2 mSv a year [12]. The natural radiation levels vary depending on the location on Earth’s surface, ranging from 1.5 to 26 mSv per year [13]. Exposure to radiation increases the risk for cancer and potential acute radiation illness beyond the protective shield of Earth’s magnetosphere. The legal limit for a radiation worker is approximately 20 mSv per year. The limits for astronauts in LEO are currently set at 0.5 Sv per year. This limit gives an estimated 3% enhanced lifetime risk of cancer [14]. The limit of 2 Sv is marked as the onset of severe radiation sickness, causing 35% fatality after 30 days. The radiation level of approximately 10 Sv causes severe radiation sickness, which is fatal in all cases within 7 days. LEO exposes astronauts to higher levels of radiation as well as altered geomagnetic and electrical fields. This requires effective shielding methods (both active and passive), available onboard radiation shelters, and improved propulsion systems to shorten the transit time to destinations beyond LEO. As of today, there is no definitive relation between the effects of space radiation exposure and astronaut morbidity and mortality [13]. However, when looking at the number of deaths due to cardiovascular diseases (CVD) in LEO astronauts and Apollo lunar astronauts (who have travelled outside of the LEO) compared to non-flight astronauts, the numbers were almost four times greater for LEO astronauts and almost five times greater for Apollo lunar astronauts, with findings of the Apollo lunar astronauts being significantly different from the LEO astronaut group (*p* ≤ 0.1). This difference in CVD risk is thought to be because of Earth’s magnetosphere, which deflects lighter GCR particles and less energetic particles away [15].

### 2.3. Microgravity

The absence of gravity removes several physical factors such as convection, buoyancy, and sedimentation. Many of these processes affect body functions and could cause clinical problems (Table 1). In the absence of convection, there is no dissipation of body heat, no sweat evaporation, and it can cause a disturbed perception of temperature in the lower extremities. In the absence of buoyancy, fluids of different density, such as water and air, do not separate in a layered manner; rather, the air bubbles remain suspended in the water, requiring an external acceleration force to separate the fluids. In the absence of sedimentation, particles remain evenly suspended in the fluid. This can affect the function of the otoliths in the inner ear in providing adequate information on the body position [12].

Upon entering µ*g*, the absence of the gravity vector decreases the hydrostatic pressure, and body fluids are redistributed toward the upper body and head (µ*g*-induced cephalad fluid shift) [12]. In addition, µ*g* drastically reduces physical activity levels in space. The upward fluid shift results in an increased vascular volume and stroke volume (SV) that distends the central vasculature, triggering the central carotid, aortic, and cardiac receptors that enable mechanisms to reduce the perceived fluid overload [36]. The distension of the heart increases the release of atrial natriuretic peptide (ANP) and stimulates baroreceptors in the carotid and aortic arteries that, in turn, inhibit the renin–angiotensin–aldosterone system [37]. Together, these responses result in a 10–15% reduction in blood plasma volume [2]. ANP also induces vasodilatation, and therefore, short-term µ*g* exposure (up to 10 days) causes vasodilatation and an acute change in vascular permeability that contributes to a decrease in plasma volume and helps to decrease atrial pressures [38]. Other mechanisms that aim to decrease the perceived fluid overload are increased diuresis or natriuresis. Apparent diuresis is not observed during spaceflight, and the reductions in blood plasma volume are believed not to be the result of increased diuresis and natriuresis but rather result from the transient fluid shift from the intravascular compartments to the intracellular spaces [39]. This results from the reduced interstitial pressures and increased upper body vascular pressures, which are characterised by typical symptoms such as ‘puffy’ faces, ‘stuffed’ noses, and ‘chicken legs’ [2]. A chronic adaptation to µ*g* is the ongoing deficit in the effective blood volume [38,40].

## 3. Space Analogues

### 3.1. Isolation and Confinement Analogues

Space analogue research has shown that human psychology and physiology are significantly altered by isolation and confinement [41]. Ning et al. [42] demonstrated in a population-based study that social isolation (both objective and the perception of social isolation) is correlated with a higher risk of mortality and a clear risk factor for developing CVD. The effects of loneliness and social stress are chronic and develop over time. The proposed underlying mechanisms are chronic overactivation of the sympathetic nervous system and physical inactivity. There are numerous sources of stress during spaceflight (Section 2), such as isolation, confinement, and separation from Earth. However, noise and vibrations associated with normal vehicle system operations or fear of equipment failure are also consistent sources of stress [43]. These stressors continue throughout the mission and may be exacerbated by interpersonal stressors and homesickness [44].

Ground-based isolation studies, including Mars105, Mars500, NEEMO (the National Aeronautics and Space Administration’s [NASA’s] Extreme Environment Mission Operation), and Antarctica missions have investigated various physiological and psychological aspects related to long-duration human spaceflight [45]. The Mars500 psychosocial experiment simulated a manned spaceflight mission to Mars that consisted of a crew of six people that spent 520 days in isolation and confinement [46]. The Mars500 study showed significant disruptions in circadian heart rate (HR) and heart rate variability (HRV). The circadian rhythm is characterised by a sympathetic predominance during the waking periods and a parasympathetic predominance during the night. The Mars105 study showed a reduced mean HR during the daytime compared with the night-time measurements during sleep, emphasising increased parasympathetic activity during the waking periods [41]. The same results were observed in the aquanauts from the NEEMO missions. Koutnik et al. [47] demonstrated decreased HR together with increased parasympathetic and reduced sympathetic modulation.

### 3.2. Radiation Analogues

The most common reported radiation-induced CVD (RICVD) included valvular heart disease, cardiomyopathy, conduction abnormalities, pericarditis, and coronary artery disease [48]. Epidemiological data from patients with breast cancer receiving radiotherapy with doses below 2 gray (Gy) to the heart showed RICVD [49]. There was a significantly increased risk of RICVD in a cohort of Russian emergency workers of the Chernobyl accidents who were exposed to doses as low as 1.15 Gy [50]. The Hiroshima–Nagasaki Life Span Study studied the cardiovascular morbidity and mortality in over 86,000 Japanese atomic bomb survivors. The radiation from bombs was composed of gamma rays and resulted in absorbed doses of 0–4 Gy in survivors. The study showed a significant increase in the risk for heart diseases such as myocardial infarction and CVDs of 14% per Gy exposure [33]. An update to the study demonstrated that the most common late effects of radiation exposure were ischaemic heart disease and hypertension [51]. Mortality from CVD, 40 years after whole-body, high-dose exposure, was significantly increased [52]. Investigations into the biological effects of radiation make use of particle accelerator facilities to test the effects of radiation on different environments, different organic materials, equipment, and technologies to understand the radiation risks, to verify functioning in space and to find appropriate countermeasures [53]. The GSI Helmholtz Centre for Heavy Ion Research (Darmstadt, Germany) has been selected by the European Space Agency (ESA) to perform research in radiobiology. In space, astronauts are exposed to charged particles ranging from Z = 1 (hydrogen) to Z = 28 (nickel). However, the probability of a hit to a single cell in the human body is low, and approximately 50% of the human cells are hit by particles of the carbon–nitrogen–oxygen group during 1 year of travel to Mars during solar minimum. Particle microbeams can deliver single charged particles of different charge and energy to single cells, therefore improving current risk estimates for long-term space travel [54].

### 3.3. Microgravity Analogues

Throughout the last six decades, various researchers have focussed on the µ*g* environment and how it affects the human organism. Nonetheless, the main difficulty while conducting these experiments is the elevated costs of launching and sustaining a human being in outer space. Given that µ*g* research is fundamental to reveal and acknowledge the impact of gravity on organisms and their biological processes [55], scientists have used and continue to operate specialised systems to decrease or avoid the launching costs and to experience directly or to simulate a similar state to the one in outer space.

**Figure 1:**
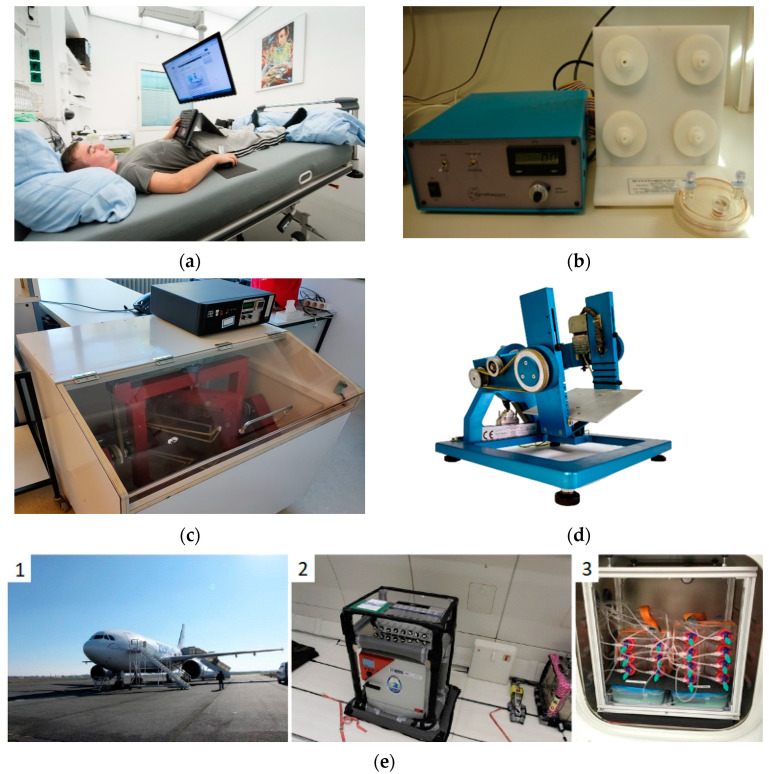
An overview of microgravity analogues. (**a**) The head-down bed rest experiment configuration (taken from the National Aeronautics and Space Administration (NASA) and Deutsches Zentrum für Luft-und Raumfahrt (DLR), CC BY-NC-ND 3.0 [56]); (**b**) a commercially available rotating wall vessel; (**c**) a commercially available three-dimensional clinostat; (**d**) a commercially available desktop random positioning machine; and (**e**) experimental setup for a parabolic flight, where image (1) shows the Airbus 310 aircraft used for the parabolic flight, picture (2) shows the incubator used for the experiment, and image (3) gives an impression of the inside of the incubator prior to take off of the flight. The images of (**e**) were published by Nassef et al. in [57].

This section lists the principal µ*g* analogues (Figure 1) used by scientists in the last decades, with a classification between in vivo and in vitro experiments.

#### 3.3.1. In Vivo Microgravity Analogues

The most common in vivo ground-based analogues to simulate µ*g* are horizontal bedrest (HBR), head-down bed rest (HDBR), water immersion (WI), and dry water immersion (DWI). 

HDBR is the most accurate bed rest model and the model of choice because its observations of headward fluid redistribution (due to a −6° angle of the bed) (Figure 1a) exceeds those observed in HBR (without an angle) [58]. HDBR studies have long been used as a spaceflight analogue to simulate the physiological changes related to spaceflight that occur due to weightlessness. The study objectives are to investigate the physiological changes induced by HDBR and to identify effective countermeasures. The −6° angle produces a gravitational force of approximately −0.1 Gz [= sin (−6°)]. This µ*g* analogue is characterised by immobilisation, inactivity, confinement, and elimination of gravitational stimuli. The induced effects are the upward fluid shift, unloading of the body’s upright weight, the absence of work against gravity, reduced energy requirements, and a reduction in overall sensory stimulation [59].

The main similarities observed between a µ*g* environment and HDBR studies are diminished bone mineral density, altered bone architecture measured by quantitative computed tomography, increased calcium in the urine, and increased risk of renal stone formation [37]. These findings have provided scientists with a method on Earth to observe how the human body adapts to µ*g* and to help design possible alternatives or treatments to improve astronauts’ health during and after space flight.

Nonetheless, it is relevant to highlight that HDBR does not offer a precise simulation of space flight weightlessness, as its subjects still experience Earth’s gravity [60]. Instead, HDBR establishes an analogous environment for the body to develop similar alterations such as the ones experienced in weightlessness. HDBR simulates the body’s reaction to µ*g* conditions in terms of arterial pressure (AP) and fluid shifts. Experts have concluded that a jump training routine, prior to HDBR, reduces the effects on the cardiovascular deconditioning provoked by 2-month inactivity with a bed rest study [61]. Another example of HDBR analysis is the one conducted by Amirova et al. [62]: they determined that changes in total peripheral resistance (TPR) and systolic blood pressure (SBP) were more pronounced after HDBR than after dry immersion. However, weight-bearing, tissue fluid redistribution and skin surface areas of compression in bed rest studies differ substantially from the ones found in weightlessness [60].

WI takes advantage of the neutral buoyancy of the human body, setting three circumstances: support withdrawal, local load elimination, and the proximity of biomechanical conditions of motor activity organisation to those in a µ*g*/weightlessness state [63,64]. These circumstances have been fundamental for scientists to choose WI as a model to test and train astronauts. Scientists have also designed the dry water immersion (DWI) setting to allow long-term experimental settings and to avoid possible complications in patients after being underwater for extended periods of time [63]. In a DWI experiment, the subjects are inside a waterproof fabric that keeps them dry during the research—hence the name. The water temperature is thermoneutral (34–35 °C), and the subjects spend 3–21 days immersed in bathtubs that support the body evenly without pressure points [58]. This method aims to simulate fluid redistribution, vascular volume changes, and post-simulation OI without medical complications such as decubitus ulcers [63].

Researchers have found that DWI subjects experience similar responses to the ones endured by astronauts during their first week in the ISS [63]. These DWI findings have helped engineering and medical teams to develop and test countermeasures such as the ‘Centaur’ and ‘Penguin’ suits, which imitate the effects of gravity on the body of the astronauts and reduce its effect on the body (e.g., the cardiovascular system) [63]. A 3-day DWI experiment showed that the subjects experienced a slight decrease in the SBP and diastolic blood pressure (DBP) (≈5 and ≈3 mmHg, respectively) [65], which is a possible contradiction to what Amirova et al. [62] found. Overall, WI and DWI experiments are mainly used to mimic µ*g* alterations on the sensorimotor system of the human body [58,63,66].

#### 3.3.2. In Vitro Microgravity Analogues

The most common in vitro ground-based analogues to simulate µ*g* are the rotating wall vessel (RWV), the three-dimensional (3D) clinostat, the random positioning machine (RPM), the drop tower, and parabolic flights.

The RWV was designed and developed by NASA to decrease the adverse effects produced by the conventional reactors [66,67] (Figure 1b). Fluid turbulence and shear effects on the cells caused by excessive agitation induce stress on the cells. Stressed cells could directly affect the results of an experiment. The RWV decreases those factors considerably [67].

As suggested by its name, the rotating wall vessel is a cylinder that contains the samples and rotates at a certain speed with its rotation axis parallel to the ground. While it may appear to be a simple machine, it applies a complex principle. 

The RWV does not allow interaction between gases and liquids. The vessel is filled with a culture medium, avoiding the turbulence caused by the continuous movement of air bubbles in similar systems. The oxygenation of the samples occurs with an axial oxygenator that supplies the oxygen in enormous quantities along with its axial structure. This process avoids bubble formation while using only dissolved gases for the exchange [68].

In addition, the continuous rotation of the vessel induces a state of perpetual fall for the cells cultured in it. Therefore, scientists also use it as a system to simulate µ*g*. The µ*g* state is directly dependent on the weight of the cells, the medium’s viscosity, and the vessel’s angular velocity [69]. Hence, every experiment that uses the RWV needs to customise the angular speed of the vessel according to the experimental set-up.

The 3D clinostat is an evolution of the two-dimensional (2D) clinostat that had been used for several decades for µ*g* simulation. In the early twentieth century, the 2D clinostat served as a test system to determine plant and animal physiology [70]. When rotating a sample with a specific velocity and orientation, the gravitation can be ‘neutralised’ [70]. During the Space Race era, experts used the 2D clinostat to experiment and corroborate data collected from space missions. During this time, scientists considered the 2D clinostat to be one of the standard practices required before sending an experiment to space [71].

Experts have improved the clinostat concept and have added one rotation axis, creating the 3D clinostat (Figure 1c). The objective of the 3D clinostat is to average the gravity vector on the sample, narrowing it as close to zero as possible [72]. With this average in time, the 3D clinostat can reach maximum values of G at the outer perimeter of the observed area [72,73,74]. The G value corresponds to the average gravity on Earth.

The 3D clinostat would rotate with a set velocity determined by the researchers. Three-dimensional (3D) clinostats usually have either a fast rotation mode or a slow rotation mode. The fast rotation mode can reach 15 revolutions per minute, while the slow rotation mode turns at a rate of 0.3 revolutions per minute. It is important to emphasise that 2D and 3D clinostats rotate at a constant angular velocity and direction. Once the experiment starts at a determined revolutions per minute, it will not change until the 3D clinostat stops. This clarification is essential to understand the next µ*g* system.

Scientists developed the RPM while perfecting the µ*g* simulation achieved with the clinostats during the twentieth century. Researchers in Japan used a 3D clinostat for plant research during the 1980s and 1990s [75]. The main difference compared with a classical clinostat was that this one rotated with a random rather than a constant velocity. Later, Dutch Space developed a similar machine, this time with an arbitrary rotation velocity and directions. This design principle gave the RPM its name (Figure 1d).

Given the random rotation of the two RPM axis, the sample (located in the crossing points of these axes) is reoriented repeatedly with respect to a static frame. This movement averages the gravity vector induced on the sample to values close to zero. However, the influence of the RPM depends directly on the position of the sample within the system. The further the sample is from the centre of rotation, the greater the gravity value experienced by it [76]. Although the RPM has been used in various research, scientists have expressed scepticism towards the simulated µ*g* obtained with the machine. An example of this is the conclusion of Brungs et al. [77], who did not recommend the random positioning and random velocity of the RPM but instead suggested using the clinostat mode of the machine (only one rotation axis at a constant velocity of 60° per second). This conclusion results from comparing the differences between parabolic flight, clinostat, and RPM conditions for the same experiment.

Nonetheless, critical reviews on the RPM have also concluded that it is ‘an ideal tool for preliminary tests, screening studies in which simulated µ*g* effects are checked on various organisms and hardware testing’ [76]. This conclusion means that the RPM is suitable for experiments before research in weightlessness, allowing scientists to prepare or change their experimental setup before a more crucial experiment.

The drop tower is one of the simpler µ*g* simulation systems. It consists of a high-altitude tower and a drop system that releases the experiments in a controllable free fall. The height of the drop towers varies around the world; some examples are NASA’s drop tower at 24.1 m [78], the Bremen tower at the Center of Applied Science and Microgravity (ZARM) at 120 m [79], and the Japan Microgravity Center (JAMIC) tower at 500 m of free fall [80].

The experiments experience a µ*g* environment during the controllable free fall. Samples inside the capsule can reach from 2 × 10^−4^ to 1 × 10^−6^ G depending on the height and location [81]. Therefore, the time of the µ*g* experiment is directly proportional to the tower’s height, varying from 2.2 s at NASA’s facility [78] to 10 s at JAMIC [80]. Although the drop tower offers a relatively inexpensive µ*g* environment for experimentation, its brief simulation time makes it feasible only for short-term research. Nonetheless, drop towers have proved to be one popular option among µ*g* experts.

Fritz and Heinz Haber suggested parabolic flights as a simulated µ*g* method while working at the department of space medicine in the United States of America [82]. Their study, which occurred during the Space Race, gave parabolic flights the recognition of experts who wanted to test and evaluate experiments in a µ*g* environment (Figure 1e).

As its name indicates, a parabolic flight consists of an aircraft that elaborates an ascending parabola. The trajectory described by the parabola needs to have specific standards to achieve high-quality µ*g*, which also depends on the aircraft used. The µ*g* environment occurs when the sum of all forces acting on the aircraft, apart from gravity, reaches values near zero [83]. Researchers can experiment on gravity levels between 1 × 10^−2^ and 1 × 10^−3^ G between 20 and 30 s [83,84,85,86,87]. This parabola is repeated consecutively to increase the timeframe of µ*g*.

An example of an aircraft that performs parabolic flights is the Airbus A300 used in Europe: after a horizontal path, the aircraft ascends with an angle of 47° for 20 s, inducing a hypergravity environment between 1.8 and 2 G. After this, pilots reduce the engine power between 20 and 25 s, allowing researchers to perform their simulated µ*g* experiments while the aircraft reaches its maximum altitude and starts descending at 42°. The last phase again induces a hypergravity environment between 1.8 and 2 G.

Several experiments have used parabolic flights to investigate the effects of hypergravity and µ*g* conditions on cardiovascular and cardiorespiratory systems. An example of these experiments is the one carried by Chiaki N. Mukai and his team. They documented the haemodynamic responses occurring while a space transport system (the Space Shuttle at the time) changes from hypergravity to µ*g* [88]. They also determined the haemodynamic relationships among four crew postures (i.e., supine, semi-supine, standing, and sitting) [88].

During another parabolic flight, researchers tried to determine the behaviour of the internal jugular pressure and its possible causality on visual impairment and intracranial pressure observed in astronauts onboard the ISS [86]. The findings revealed that the internal jugular venous pressure increases considerably with fluid shifts induced by µ*g*. However, there were no direct correlations between the internal jugular venous pressure and the visual impairment and intracranial pressure.

Parabolic flights offer a relatively fast opportunity to experiment on µ*g*. Notwithstanding its short period, the µ*g* environment generated allows researchers to make real-time changes according to the needs and is one of the first steps before a space experiment.

## 4. Cardiovascular Diseases Related to Microgravity (In Vivo)

The ISS is one of the most famous systems for outer space ever built. The ISS is a joint work between five space agencies that took 10 years and more than 30 missions to assemble [89].

Inside the ISS, there is no zero gravity as people tend to believe. Instead, the environment experienced onboard the ISS is affected by low-level accelerations and vibrations. These forces result from the ISS’s free fall due to its orbital flight and the design of the ISS itself [89]. The lowest possible gravity (acceleration) is located in a floating point close to the ISS centre of mass [89].

The ISS has 16 pressurised modules (habitable for humans): nine American, four Russian, two Japanese, and one European. The ISS comprises modules, and all elements attached to the structure of the space station experience vibrations from multiple sources (e.g., fans, pumps, atmospheric drag, and attitude control system) and are transmitted by the ISS structure itself (mechanically) and the air (acoustically) [89].

Inside the ISS, the spectrum of the vibrations oscillates between the steady frequencies to 300 Hz. Nonetheless, these vibrations possess different magnitudes, ranging from 1 × 10^−5^ m/s^2^ at low frequencies to 1 m/s^2^ at high frequencies [89]. Given that the lower acceleration levels are approximately one millionth of the Earth’s gravity (1*g* ≈ 9.8 m/s^2^), the environment inside the ISS is considered to be µ*g*.

Approximately 45% of the experiments that are part of ISS research concern human physiology and biology [90]. In general, around 3000 µ*g* science experiments have occurred in the 20-year ISS history (2000–2020). From these, more than 1200 have focussed on biological and biotechnological behaviour in this environment [91]. The main participants are the Japan Aerospace Exploration Agency (JAXA), NASA, and Roscosmos; however, the ESA is also a contributor [91].

The ISS offers scientific capabilities, providing a unique laboratory to investigate life or biological sciences without the effects of gravity [92]. Public and private industries use these investigations to improve medical techniques, pharmaceutics, food supplies on Earth, and upgrade the life-support capabilities for space exploration. Given the vast number of experiments, this review does not pretend to enumerate all the in vivo experiments carried onboard the ISS. However, some of the most well-known results found during the last decade are discussed in the next section.

In addition to the ISS, satellites such as Bion-M and Foton-M are also available. Bion-M is a specialised satellite developed by joint work between international space agencies for space medicine and biology studies [93]. It came from the Bion programme, which was initially designed and operated by the Soviet Union. Nonetheless, at the beginning of the twenty-first century, Russian scientists redesigned the satellite to fulfil new standards for space experimentation [94]. Similar to the Bion-M, the Foton-M project improved a previous enterprise, the Foton satellites, which studied material sciences and biology [95]. The first Foton satellites had an orbital life-span of around 15 days, but the Foton-M4 had a design to maintain its orbit for at least 2 months [95].

### 4.1. Cardiac System Adaptations and Remodelling

#### 4.1.1. Haemodynamic Adaptations

**Figure 2:**
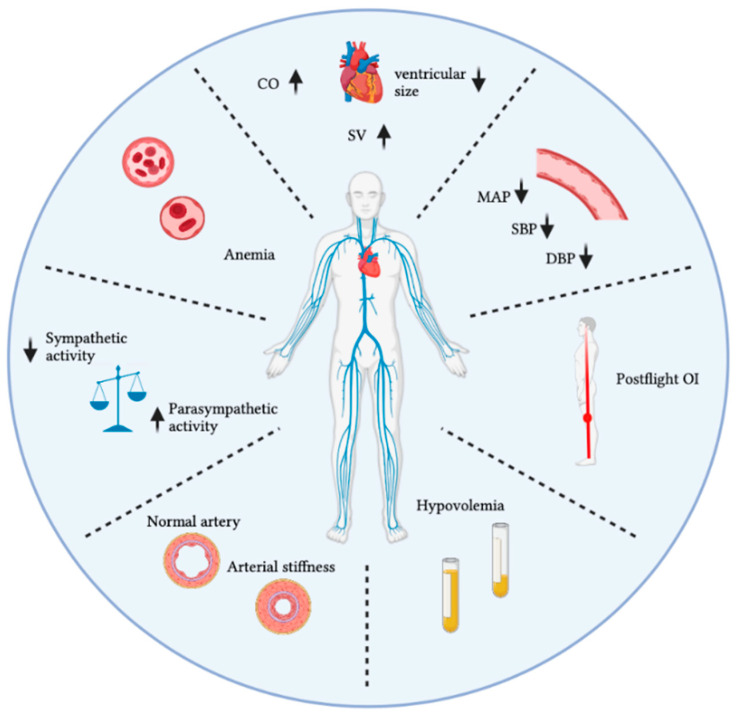
A summary of spaceflight-induced cardiovascular diseases. Abbreviations: cardiac output (CO); stroke volume (SV); mean arterial pressure (MAP); systolic blood pressure (SBP); diastolic blood pressure (DBP); orthostatic intolerance (OI); increase (↑); decrease (↓).

Upon entering µ*g*, there is an initial increase in vascular volume and stroke volume (SV) resulting from the upward shift of bodily fluids. In the first 24–48 h of spaceflight, this µ*g*-induced cephalad fluid shift leads to cardiac distension, increasing the size of the heart chambers by 20% due to the rise in left ventricular volume [38]. Approximately 2 L of fluid is shifted towards the upper extremities and head, simultaneously increasing the cardiac output (CO) by 18%–26% [17,18]. Prisk et al. [19] studied the short-term effects of µ*g*-induced fluid shift on the CO and SV. The CO increased by 18% inflight and decreased by 9% postflight compared with preflight standing measurements. In addition, the SV increased by 46% inflight and decreased by 14% postflight compared with preflight standing measurements [19]. The elevation in CO is induced by a rise in SV because the HR remains either unchanged or is slightly reduced. The increase in SV results from the rise in cardiac preload induced by the upward fluid. Norsk et al. [19] examined CO changes during spaceflight and demonstrated that a long-duration spaceflight (3–6 months) increases SV by 35% and CO by 41%, which contradict earlier findings of decreased or unchanged SV and CO by Herault et al. [94]. Furthermore, researchers have reported a relationship between the average SV and sympathetic nerve activity: a decrease in SV induces an increase in sympathetic nerve activity [20,96]. A summary of the spaceflight-induced CVD can be found in Figure 2.

#### 4.1.2. Structural Adaptations

The cardiac muscle adapts well to changes in loading conditions. After the initial elevation in left ventricular volume, there is a decrease in ventricular size. In the µ*g* environment, less contractility of the heart is required to send blood towards the head and maintain AP [97]. Blomqvist [21] showed a 10% decrease in ventricular size after 24–48 h inflight compared with preflight measurements. In addition, two studies showed a 9%–23% decrease in left ventricular size postflight compared with preflight measurements [22,98].

Perhonen et al. [23] showed that the heart atrophied by 8%–10% after a 10-day spaceflight. Cardiac atrophy likely results from µ*g*-induced reductions in metabolic demand and oxygen uptake [98]. In addition, during spaceflight, the configuration of the heart changes from being more elliptical on Earth to more spherical in space. The mean spherical index changed by 9.4%—from 2.01 on Earth to 1.82 in µ*g*—a finding that verifies the anatomical change of the heart in space [99]. The heart shape and left ventricular mass returned to normal form 3 days postflight [98].

#### 4.1.3. Cardiac Arrhythmias

Various occurrences of arrhythmias have been noted throughout human spaceflight. According to Vernice et al. [100], they appear to be frequent, albeit transient. Commonly reported arrhythmias include atrial and ventricular premature contractions (VPCs), short-duration atrial fibrillation, and non-sustained ventricular tachycardia.

The Gemini and Apollo missions showed occasional premature ventricular contractions and atrial premature contractions (APCs). One astronaut during the Apollo 15 mission had bigeminal VPCs and APCs due to a decrease in total body potassium level [12,100]. During the Skylab programme, there were several reports of VPCs and APCs. One astronaut showed multifocal VPCs after performing an extravehicular activity. The proposed causes were decompression, low fluid intake, psychological stress, and physical exertion [100]. The Russian space agency reported a total of 75 arrhythmias and 23 conducting disorders after the Mir era [100]. The specific origin of cardiac arrhythmias remains unclear. Several proposed causes of cardiac arrhythmias include hypercapnia (increased levels of CO_2_ in isolated and enclosed environments, such as the ISS), hypokalaemia (decreased levels of potassium in the blood), physical and emotional stress, poor nutrition with a potential impact on electrolyte concentrations, chronic atherosclerosis (AS) causing a poor blood supply, or medical therapeutics that influence electrocardiography (ECG) parameters [12].

### 4.2. Vascular System Adaptations and Remodelling

#### 4.2.1. Haemodynamic Adaptations

In µ*g*, the blood is distributed uniformly throughout the body, and AP is uniform from the head to the feet because the gravitational force and gradient along the longitudinal direction of the body is zero. Within the first 24 h of spaceflight, despite the increase in the central blood volume upon entering µ*g*, astronauts experience a decrease in central venous pressure (CVP) [27] and DBP (5 mmHg), while their SBP and mean arterial pressure (MAP) remain unchanged [24]. Early on during spaceflight (4–12 days), there is an observed decrease in lower limb blood flow together with an increase in vascular resistance [28]. The decrease in CVP has been observed simultaneously with the increase in left ventricular end-diastolic volume [25]. Parabolic flight experiments have demonstrated a 1.3 mmHg decrease in CVP, while the atrial diameter increased by 3.6 mm [26]. The loss of gravitational compression on the thoracic cage and mediastinum may induce the CVP to decrease due to the reduction in intrathoracic pressure [101]. By contrast, Norsk et al. [20] demonstrated a decrease in average SBP (8 mmHg), DBP (9 mmHg), and MAP (10 mmHg) during long-duration spaceflight (3–6 months), which is accompanied by a decrease in systemic vascular resistance of 39%. The nightly blood pressure dip of 8 mmHg was maintained in space. The decrease in systemic vascular resistance is indirectly deduced from a measured decrease in blood pressure and an increase in CO, despite the preserved or increased sympathetic nerve activity [29]. Nicgossian et al. [102] reported that the baroreceptor function is changed significantly in astronauts from preflight to days 2–3 after returning to Earth. The baroreceptors of postflight astronauts will react to a higher change in carotid distending pressure and will promote a smaller rise in HR than the baroreceptors of the astronauts preflight [102]. 

#### 4.2.2. Structural Adaptations

Ground-based analogues, simulating µ*g*, have demonstrated a decrease in vascular smooth muscle constriction ability, adrenergic responsiveness, and alterations in nitric oxide (NO) physiology [103]. It has been proposed that the increase in biomarkers of oxidative and inflammatory stress during long-duration spaceflight is related to changes in long-term vascular structure and function, which is assessed by the carotid intima media thickness (IMT) and flow-mediated dilatation. After 6 months of spaceflight, the diameter of the femoral and carotid artery remained unchanged, but the IMT rapidly increased [104]. Carotid IMT increased up to 10–12% after 6 months of spaceflight and up to 20% after 1 year on the ISS [31,104]. The femoral IMT increased by approximately 10–15% after 6 months of spaceflight [104]. Hughson et al. [33] demonstrated a decrease in pulse transit time, suggesting an increase in central and peripheral arterial stiffness. The carotid artery distensibility coefficient and the beta-stiffness index reflect a 17–30% increase in arterial stiffness postflight compared with preflight measurements [34]. Other studies have also demonstrated that the carotid and femoral arterial stiffness increased after 6 months of spaceflight [33,35]. Navasiolava et al. [35] reported a slight decrease in upper limb blood flow and an impaired endothelial dependent vasodilation after 3 weeks of spaceflight.

To summarise, the observed changes have revealed an increase in IMT and vascular stiffness and a decrease in distensibility [32,104]. The initial mechanisms of increased arterial stiffness and resistance are cephalad fluid shift and the loss of hydrostatic pressure gradients (Figure 2). Carotid arterial stiffness recovered to normal by the fourth day after return to Earth [38].

### 4.3. Endothelial Dysfunctions and Atherosclerosis

Spaceflight is associated with a number of cardiovascular risk factors such as changes in normal exercise routine, altered dietary habits, increased psycho-social stressors, and elevated radiation exposure. All of these changes might promote oxidative stress and inflammation that could impair endothelial cells (ECs) and might accelerate the development of CVD [105]. 

The endothelium plays an important role in the regulation of microvascular homeostasis and blood flow [106]. The tunica intima is the innermost layer of the blood vessels—both arteries and veins—and consists of a monolayer of ECs that can modulate arterial stiffness by releasing vasoactive agents that modulate vessel tone [107]. When the blood pressure is low, ECs secrete various vasoactive molecules (i.e., angiotensin II, endothelin-1, and reactive oxygen species (ROS)) that act on the vascular smooth muscle cells (VSMCs) to promote vasoconstriction [108]. When blood pressure increases, vasodilator substances are produced by ECs (i.e., NO, prostacyclin, and endothelium-derived hyperpolarising factor) [109]. Endothelial dysfunction is a proposed result of spaceflight-induced ROS increase that limits the bioavailability of NO. This compound relaxes smooth muscle cells and ensures vessel patency. Damaged or excessively activated ECs can secrete vasoconstrictor factors as well as factors that affect the differentiation and growth of VSMCs [110]. ROS also act as secondary messengers and can increase intracellular Ca^2+^ concentrations, further promoting vasoconstriction [111]. 

Neural and hormonal factors might also play a role in the increase in arterial stiffness [112]. Although there is a decrease in peripheral vascular resistance, there is evidence for elevated circulating catecholamine levels and an increase in vasomotor tone and muscle sympathetic nerve activity, which is directly measured from peroneal nerve sympathetic activity [40]. Angiotensin II and aldosterone concentrations are increased during spaceflight and induce collagen formation, matrix remodelling and hypertrophy, the proliferation of VSMCs, and eventually endothelial dysfunction [113,114]. 

Ground-based simulations of µ*g* have been conducted to explore the effects of pro-oxidative environments. Overall, oxidative stress is implicated in the pathophysiology of CVD. An imbalance between ROS and antioxidants increases the risk of oxidative damage and inflammation. An abundance of ROS can damage cellular components such as lipids, proteins, and DNA [115]. Lee et al. [105] demonstrated elevated levels of oxidative stress and inflammatory biomarkers during spaceflight. These levels returned quickly to preflight levels upon return to Earth [105]. Oxidative stress is important in the pathogenesis of AS. Impaired arterial function and thickening of the carotid arteries are two sub-clinical indicators of the development of AS. AS is associated with the build-up of an atheromatous plaque. This mainly consists of oxidised low-density lipoprotein (LDL) and macrophages inside the artery walls [116]. Total cholesterol and LDL are elevated during spaceflight but return to preflight levels shortly after spaceflight. In the progression of AS, the differentiation of stem cells to VSMCs contributes to the increased IMT. However, these mechanisms are currently unknown and speculative [117].

### 4.4. Blood Composition

Initially, the red blood cell (RBC) concentration increases as the plasma volume decreases. This will prompt the body to decrease the production of new RBC (erythropoiesis) to re-establish homeostatic balance. Short-duration space missions (10–14 days) have demonstrated an average 10–15% decrease in haematocrit, which is measured immediately after landing. This corresponds to a decrease of 1% RBC mass per day in space [2]. Erythropoietin (EPO) is a glycoprotein cytokine that is produced by the kidneys and stimulates RBC production in bone marrow. A decrease in reticulocyte count (immature RBC) and a decrease in serum EPO levels might indicate a change in erythropoiesis [38]. Udden et al. [118] concluded that over a 9-day mission, there was a reduction in RBC mass because few new RBC were released from the bone marrow. Another study also demonstrated a decrease in EPO levels in µ*g*. Upon return to Earth, the EPO levels increased twofold compared with preflight measurements [119]. Interestingly, Kunz et al. [16] showed an increase in RBC concentrations during long-duration spaceflight, suggesting that the body acclimates to µ*g* conditions and spaceflight anaemia might be less of a problem than initially thought.

### 4.5. Autonomic Cardiovascular System Adaptations 

The autonomic nervous system is a control system that plays a key role in regulating various physiological functions, such as HR, blood pressure, and peripheral resistance of blood vessels. Autonomic cardiovascular control can be measured in a non-invasive way from the HRV and blood pressure variability [38]. HRV entails changes in the time interval between consecutive heart beats. The time interval fluctuations are complex and allow the cardiovascular system to respond to the various physical and psychological needs of the body. The HRV frequency domain values calculate the distribution of absolute or relative power into four frequency bands (HR oscillations can be divided into ultra-low frequency, very-low frequency, low frequency, and high frequency). The low frequency (LF) to high frequency (HF) ratio (LF/HF) might estimate the dynamic ratio between the sympathetic nervous system and parasympathetic nervous system [120]. 

Spectral analysis of HRV has demonstrated a reduction in inflight LF power, while the HF power remained unchanged compared with preflight conditions. Another study demonstrated similar LF/HF ratios and HF values inflight compared with preflight supine conditions. This indicates a predominant vagal control of HR during spaceflight. A recent study on the ISS demonstrated a decrease in the LF component of HRV, while the HF component remained unchanged during short-duration missions, again demonstrating vagal predominance [121]. It is also shown by Migeotte et al. that the HRV of crew members in space was significantly decreased during microgravity compared to preflight for normal breathing crew members in both supine and standing position, but postflight, the HRV was increased significantly in normal breathing standing crew members compared to inflight, while it was increased insignificantly for normal breathing supine crew members [30]. 

Over the last 40 years, there have been conflicting results regarding whether inflight HR is altered compared with preflight data [122]. However, recent research has concluded no significant changes from preflight resting rates [121]. The highest rates have been noted upon return to Earth, immediately after spaceflight [122]. Interestingly, the most prominent increase in postflight HR has been reported after very short-duration space missions (4–5 days) [22].

### 4.6. Orthostatic Intolerance

On Earth, changes in posture result in the generation of a hydrostatic pressure gradient along the longitudinal direction of the body due to the force of gravity. The hydrostatic pressure is low at the head and high on the feet, which is a pattern that causes higher arterial and venous pressures in the lower extremities. The increase in intravascular pressure induces vasodilatation and footward fluid shift when posture is changed from a supine or recumbent position to an upright position [97]. Consequently, footward blood shift may decrease the venous return to the heart and thus decrease the SV and CO [123]. AP is the product of CO and total peripheral resistance (TPR). To maintain AP, the HR and TPR are increased reflexively [124]. 

**Figure 3:**
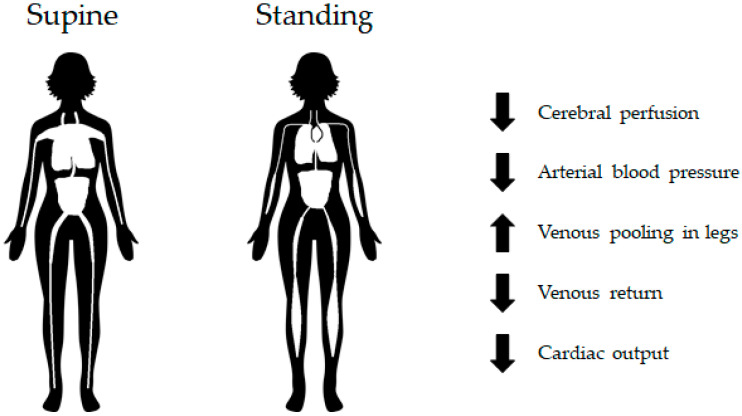
Orthostatic stress diagram. In a standing position, gravitational stress induces a downward shift in blood volume. This figure illustrates the result of cardiovascular deconditioning; that is, the inability of the cardiovascular system to maintain adequate blood pressure and brain perfusion. Increase (↑); decrease (↓).

OI is one of the main medical challenges in astronauts after return to Earth [125]. Postflight OI can be defined as the inability to haemodynamically cope with orthostatic stress. In an upright position, this is the gravitational stress inducing a downward shift in blood volume (Figure 3). The underlying mechanism of OI is spaceflight-induced cardiovascular deconditioning [38,106]. Cardiovascular deconditioning (i.e., hypovolemia, cardiac atrophy) affects the body’s ability to maintain adequate arterial blood pressure and brain perfusion in the upright position. In other words, OI is the inability to compensate for the postural decrease in arterial blood pressure and can result in syncopal or presyncopal symptoms, such as nausea, headache, dizziness, hypotension, vomiting, sweating, and fatigue. These symptoms are observed in 28–65% of astronauts performing a stand test or tilt-table test upon return to Earth [126]. It is well documented that OI in astronauts is associated with low vascular resistance and reduced vascular smooth muscle contraction [97,126]. The baroreceptor vascular dysfunction observed after spaceflight decreases vasoconstrictor responsiveness [103]. The incidence of postflight OI after short-duration missions (4–18 days) was approximately 20–40%. The incidence in postflight OI after long-duration missions was approximately 80% [1]. 

Researchers have observed a relationship between the average SV and sympathetic nerve activity. Changes in posture from a supine to a standing position induce a decrease in SV and an increase in sympathetic nerve activity [97]. The relationship between the two are similar for subjects with presyncope or without syncope upon return to Earth [127]. The central baroreflex responses to changes in SV are maintained postflight (increase in HR, tachycardia) and are similar to the responses observed preflight, despite the decrease in cardiac size and blood volume [128]. Postflight, a decrease in vascular contractility is observed (peripheral baroreflex response). Astronauts who could not complete 10 min of standing after short-duration spaceflight (9–14 days) showed a significantly reduced vasoconstrictive response [126]. It is important to note that the vascular resistance is already increased after spaceflight compared with preflight measurements. The elevated resting vasoconstriction might explain the reduced response of vasoconstriction [97,126]. 

An important distinguishing feature between astronauts who tolerated and did not tolerate the stand test on landing was the inability of the non-tolerant group to maintain MAP and increase TPR above preflight measurements [129].

## 5. Cellular and Molecular Adaptation of Cardiovascular Cells to Spaceflight (In Vitro)

### 5.1. Fibroblasts

Beck et al. [130] investigated the effects of 24 h of simulated µ*g* with the RPM on irradiated mouse foetal fibroblasts. They showed that the changes in the cell cycle caused by radiation were the same for the cells exposed or not exposed to µg, but the simulated µ*g* changed the foetal fibroblasts independently of the radiation. The authors showed that the foetal fibroblasts exposed to µ*g* induced by the RPM had significantly decreased caspase-3 activity (a measure of apoptosis) at all radiation doses compared with the gravity controls [130].

Beck et al. [131] have also investigated the effects of 65 h of simulated µ*g* using an RPM, chronic irradiation, or a combination of the two on murine foetal fibroblasts. The cells subjected to simulated µ*g* showed significant upregulation of oxidative stress response genes, such as glutathione-S-transferase α1 and α2, a modifier subunit of glutathione cysteine ligase, and haem oxygenase 1, and these genes are targets of the nuclear factor-erythroid 2 p45-related factor 2. As a result of the upregulation of these genes, nuclear factor-erythroid 2 p45-related factor 2 might have a role in counteracting oxidative stress in simulated µ*g*. Furthermore, the simulated µ*g* also significantly downregulated genes involved in cytoskeletal remodelling, such as actin gamma 2 (in smooth muscle), actin alpha 1 (in skeletal muscle), calponin 1, and four and a half LIM domains 1, and these four genes are regulated by serum response factor, which is mediated by the Rho signalling pathway. Irradiation produced the greatest effect in the gene set enrichment analysis: it decreased significantly genes involved in cytoskeletal remodelling, DNA damage response, and cell cycle regulation. The interaction between simulated µ*g* and irradiation is complex, because many of the altered genes or gene sets from the individual treatment of either simulated µ*g* or irradiation were not altered in the combined treatment of irradiation and simulated µ*g*, but the combined treatment still showed a significant downregulation of glutathione-S-transferase α1 and α2, haem oxygenase 1, actin gamma 2 (in smooth muscle), calponin 1, four and a half LIM domains 1, and in genes involved in cell cycle regulation [131].

### 5.2. Vascular Smooth Muscle Cells

**Figure 4:**
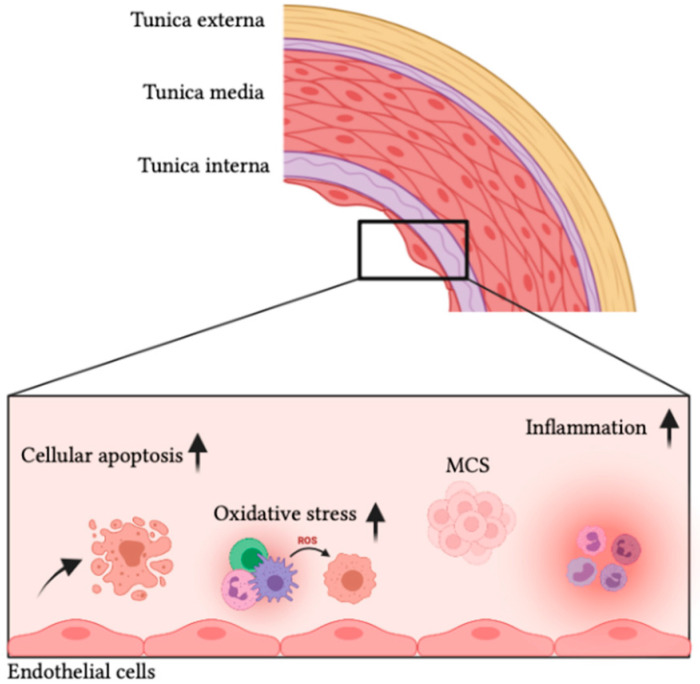
The endothelial cell response to microgravity. Multicellular spheroids (MCS); reactive oxygen species (ROS); increase (↑).

VSMCs reside in the tunica media (Figure 4), the thickest layer of the arteries, and regulate blood flow and blood pressure of the vasculature by contracting and relaxing in response to different stimuli [33,132]. Furthermore, they play a crucial role in vessel remodelling in the case of pregnancy, exercise, and vascular injury [133,134]. Under pathological conditions, VSMCs lose their ‘contractile’ phenotype, increase proliferation and extracellular matrix (ECM) production, and shift toward a ‘synthetic’ phenotype (this process is called dedifferentiation) [133,135]. Ground-based animal studies have demonstrated µ*g*-induced VSMC remodelling in the common carotid and basilar arteries in tail-suspended rats subjected to hindlimb unloading [133,136]. Kang et al. [131] concluded that simulated µ*g* suppresses VSMC proliferation and migration, enhances cell apoptosis, stimulates NO release, and damages the cellular cytoskeleton.

Extracellular Ca^2+^ influx through voltage-dependent Ca^2+^ channels (VDCCs) is one of the main components of Ca^2+^ signalling in VSMCs. The two main types of VDCCs are the high voltage activated L-type and low voltage activated T-type Ca^2+^ channels [137]. The expression of both L-type and T-type VDCCs are significantly upregulated in differentiated contractile VSMCs in rats exposed to simulated µ*g*. L-type VDCCs are reportedly decreased in dedifferentiated synthetic VSMCs and suppress the dedifferentiation of VSMCs [138]. However, Xue at al. [130] demonstrated that simulated µ*g* could upregulate the expression of L-type VDCCs in dedifferentiated synthetic VSMCs, suggesting other potential Ca^2+^ signalling pathways that are responsible for the dedifferentiation of cerebral VSMCs under simulated µ*g*. Zhang et al. [139] demonstrated enhanced T-type VDCC current through the upregulation of Ca_v_3.1 channel. Due to the Ca^2+^ influx, the Ca^2+^-sensitive calcineurin activity is activated and then translocated to the nucleus to promote VSMC dedifferentiation and proliferation [139]. µ*g*-induced vascular structural and functional remodelling may be one of the key contributors to postflight OI [140,141].

### 5.3. Endothelial Cells

ECs reside in the tunica intima (the inner most layer of blood vessels) (Figure 4) and play an important role in the regulation of microvascular homeostasis and blood flow [106]. 

On the ISS, researchers have compared ECs from on-ground experiments with a flight experiment carried out in 2012. The investigation used human umbilical vein endothelial cells (HUVECs) and examined the effects of short-term spaceflight on the growth and morphology of these cells [142,143]. After 12 days in the µ*g* environment, the ECs presented acute changes related to cytoskeletal lesions and increased membrane permeability [142,143]. In addition, the ECs did not show recovery from the cytoskeletal changes and displayed reduced metabolism and cell growth after returning to Earth [142,143].

Another study also tested the response of HUVECs to the µ*g* environment of the ISS. After 10 days, the researchers concluded that µ*g* negatively affects HUVEC functions [144]. Scientists noticed that the cells reacted as if they were in a disturbed flow, although their growth medium was stable [144]. As a result of this behaviour, HUVECs overexpressed thioredoxin-interacting protein, which is related to age-related diseases and cardiovascular disorders. Moreover, genes that alter mitochondrial processes were downregulated, thus promoting senescence of the cells [144].

#### 5.3.1. Spheroid Formation and Angiogenesis

Ground-based µ*g* studies have demonstrated that ECs exposed to the RPM grow as a 2D monolayer and form 3D structures such as spheroids—formed by spontaneous aggregation of cells—and tubular constructs [145,146]. Pietsch et al. [147] developed an automatic cell culture container in which they demonstrated that ECs form spheroids when cultured in µ*g* on the ISS. Krüger et al. [148] demonstrated similar results.

Previous studies have revealed that ECs are sensitive to variations in gravity. Researchers suggest that they convert gravitational stimuli via gene regulation into biochemical signals [146].

Dittrich et al. [149] performed a 35-day RPM-exposure experiment on ECs and investigated the proteins associated with cellular shape, angiogenesis, and endothelial dysfunction. After 35 days, there were increased levels of the ECM protein fibronectin (FN), vascular endothelial growth factor (VEGF), interleukin (IL)-6, IL-8, and vascular cell adhesion molecule 1 in multicellular spheroids (MCS). VEGF is an important regulator in angiogenesis and tube formation, whereas IL-8 plays an important role in spheroid formation in cancer and benign cells. Other factors that are involved in angiogenesis are monocyte chemoattractant protein, which is regulated on activation normal T-cell expressed and secreted (RANTES) and neutrophil gelatinase associated lipocalin (NGAL) [149]. RANTES is believed to be related to the promotion of EC migration and neo-vessel formation, while NGAL is proposed to have pathophysiological importance in the development of CVD [150,151]. All three factors were elevated in MCS after 35 days of RPM exposure. In vitro tube-like structure formation represents blood vessel growth that is controlled by several growth factors (VEGF) and cytokines (IL) [149].

#### 5.3.2. Endothelial Processes

Maier et al. [145] showed a gravity-dependent modulation of endothelial processes such as cellular proliferation, apoptosis, cytoskeletal organisation, intracellular signalling mechanisms, and growth behaviour (Figure 4). Morbidelli et al. [152] demonstrated a marked impairment in EC responsiveness to angiogenic growth factors and a reduced ability to proliferate by the upregulation of apoptotic signals. Following exposure to simulated µ*g*, porcine aorta ECs tend to change their morphology and gene expression patterns. These are believed to trigger pro-apoptotic signals, which are accompanied by mitochondrial disassembly [152]. However, these results are not observed in all ECs, such as HUVECs and dermal human mammary epithelial cells [145].

The cytoskeleton plays an important role in the adaptation of cells to mechanical stress, including changes in hydrostatic pressure. In most cells, the cytoskeleton is formed by a 3D composite network composed of actin filaments, microtubules (MT), and intermediate filaments (IFs). Together with a host of proteins that bind to the sides or ends of these polymers, these proteins regulate filament strength, crosslink filaments to each other, and apply forces to the filaments [153]. Previous research on mechanical unloading has demonstrated the remodelling of actin filaments, a reduction in the content of these filaments, and a significant effect on the arrangement of both actin filaments and MT [154,155]. Grenon et al. [156] revealed the disorganisation and clustering of actin microfilaments around the nucleus. Janmaleki et al. [157] demonstrated a 65% decrease in actin filament content and a 26% decrease in the beta-tubulin content in ECs after 24 h of simulated µ*g*. In addition, the study revealed a considerable disruption in the actin rim under the plasma membrane. MT were disorganised and relatively concentrated around the nucleus [157]. However, another study using clinostats demonstrated alterations in MT and Ifs, which were thicker and increased after 24 h of simulated µ*g*. The IF network for cytokeratin had increased and gathered in a dense centre near the nucleus. The same was observed for MT (alpha-tubulin) that were coiled in close contact with the nucleus and grew under simulated µ*g* after 24 h [158].

Grimm et al. [159] cultured ECs in the RPM and showed that together with an increase in actin content, there was an overexpression and clustering of beta-1 integrin, laminin (LM), and FN. LM and FN are involved in the regulation of cell adhesion and migration. The authors proposed that the observed changes related to exposure to simulated µ*g* cause a dysregulation in cell motility and adhesion to substrates [159].

#### 5.3.3. Arterial Stiffness

ECs can modulate arterial stiffness by the release of vasoactive agents that modulate vessel tone. Researchers recently demonstrated that µ*g* and simulated µ*g* induce an increase in human and rat arterial stiffness [33,34]. Tuday et al. [160] hypothesised that alterations in vessel wall collagen or elastin content or in ECM cross-linking could be responsible for the increase in vessel stiffness. The study demonstrated an increase in aortic wall collagen content and ECM cross-linking enzymatic activity in rats after 7 days of hindlimb unloading. The vascular wall consists mainly of type I (70–75%) and type III (20–25%) collagen, and changes in the ratio of the types of collagen in the wall vessel is believed to influence the vascular stiffness [161]. However, the collagen subtype composition and aortic elastin content were not altered by seven days of hindlimb unloading [160]. Infanger et al. [158] demonstrated that simulated µ*g* using clinostats resulted in an increase in ECM proteins, such as collagen type I, FN, osteopontin, and LM.

### 5.4. Cardiomyocytes 

Cardiomyocytes are also altered by µ*g*. Liu et al. [160] exposed the HL-1 cardiomyocyte line to µ*g* in a 2D clinostat for 48 h and compared them with a control group. There was atrophy of the cardiomyocytes and altered spontaneous calcium signalling. The altered spontaneous calcium signalling was denoted by a significant increase in basal cytosolic calcium and by an increase in spontaneous calcium oscillations after 48 h of µ*g* compared with the control group. This shows that intracellular calcium signalling can be promoted by µ*g*. After 48 h of µ*g*, the cardiomyocytes were significantly smaller and atrophied compared with the control group. Furthermore, they exhibited an increased phosphorylation of Ca^2+^/calmodulin-dependent protein kinase II δ (Thr287) and histone deacetylase 4 after 48 h of induced µg. After 48 h of induced µ*g*, there was also a significant increase in the expression of the foetal genes that encode ANP and brain natriuretic peptide, which indicates remodelling of the myocardia, a decrease in myosin heavy chain α, and thus altered cardiomyocyte structure [162].

Xiong et al. [161] used a clinostat to explore the effect of simulated µ*g* on the level of NO in rat cardiomyocytes. The cells were divided into four groups, each exposed to simulated µ*g* for a different time (8, 24, 36, and 48 h), compared with a control group. After 8 h of simulated µ*g*, NO had already increased compared with the control group. After 8 h, the NO level was 2.16 times higher than the control group, after 24 h, it was 3.02 times higher, after 36 h, it was 3.94 times higher, and after 48 h, it was 4.00 times higher [163].

### 5.5. Stem Cells 

A recent study compared neonatal and adult cardiovascular progenitor cells (CPCs) on Earth and onboard the ISS during 30 days [164]. Experts observed that neonatal CPCs had less of a capacity to form endothelial-like tubes than their adult counterparts. However, neonatal CPCs cultured on the ISS multiplied more than the adult cells and the cells grown on Earth [164]. The researchers also found an increase in transcripts for DNA repair proteins but not of the genes related to apoptosis

In 2016, researchers exposed engineered 3D progenitor cardiac spheres to simulated µ*g* [165]. This technique produced ‘highly enriched cardiomyocytes (99% purity) with high viability (90%)’. To achieve these results, the authors cultured human pluripotent stem cells (hPSCs) 3D structures on the RPM for 3 days, developing a novel culture system that increased the ‘induction, proliferation and survival of cardiac progenitors’ [165]. These parameters are generally desired when using hPSCs to obtain cardiomyocytes in the field of regenerative medicine [165].

Muscle stem cells, also called satellite cells, are affected by µ*g*; the effect of µ*g* on these cells depends on the exposure time [166]. The authors found that simulated µ*g* with the RPM increased the expression of bone morphogenic protein-2 (BMP-2) and the number of myotubes during the early phases of simulated µ*g* (≤72 h) compared with control cells in normal gravity; these changes indicate an increased activation of satellite cells. However, between 72 and 110 h (the end of the experiment), there was an increased death of newly formed myotubes and satellite cells [166].

Several studies have shown that µ*g* affects stem cells. Xue et al. [165] demonstrated that simulated µ*g* by a clinostat regulated the differentiation of mesenchymal stem cells (MSCs) from a rat, and this regulation was dependent on the amount of time spent in simulated µ*g*. After 72 h of simulated µ*g*, there was a significant increase in the neuronal, adipogenic, and endothelial differentiation of the MSCs. This was different after 10 days, at which time the simulated µ*g* had promoted a significant increase in osteogenic differentiation to osteoblasts, while the neuronal, adipogenic, and endothelial differentiation had been decreased significantly. There was also an effect on ras homolog family member A (RhoA), which is a small GTPase. After 72 h of simulated µ*g*, the level of RhoA was significantly reduced, while the level of RhoA was significantly increased after 10 days. When RhoA was inhibited, the MSCs differentiated to adipogenic cells instead of osteogenic cells after 10 days of simulated µ*g*, indicating that a low level of RhoA is associated with neuronal, adipogenic, and endothelial differentiation of the MSCs, while a high level of RhoA is associated with the osteogenic differentiation of MSCs [167].

#### Endothelial Progenitor Cells

Kong et al. [168] examined the effect of simulated µ*g* by a 3D clinostat on conditioned media (CM) of human endothelial progenitor cells (EPCs) compared with the CM of EPCs cultured under normal gravity. The proliferation of EPCs during µ*g* was significantly suppressed already after 12 h of µ*g* compared with EPCs under normal gravity, and that suppression became greater after 24 and 48 h. However, µ*g* significantly upregulated endothelial NO synthase and hypoxia-induced factor-1α after 12 and 24 h compared with normal gravity, but prolonged exposure to µ*g* significantly decreased hypoxia-induced factor-1α and insignificantly decreased endothelial NO synthase. Furthermore, there was also upregulation in the NO concentration after 12, 24, and 48 h of µ*g* compared with EPCs exposed to normal gravity [168]. In addition, the authors [168] also showed that the expression of Ki67 and proliferation of HUVECs were significantly increased by CM of EPCs cultured in µ*g* compared to CM of EPCs in normal gravity; this effect was NO-dependent. Furthermore, the expression of matrix metalloproteinase-9 and VEGF, which are angiogenic markers, were also significantly elevated in HUVECs exposed to CM of EPCs in µ*g* compared with CM of EPCs in normal gravity [168]. Ramaswamy et al. [169] reported that EPCs exposed to µ*g* for longer than 18 h had significantly reduced or no colony formation. Moreover, Kong et al. [168] also injected CM of EPCs cultured in µ*g* into rats to examine its effects on fracture healing. This CM significantly accelerated callus growth after 14 days, and the mechanical test after 21 days showed a significant improvement in ultimate load and energy to failure compared with rats injected with CM of EPCs cultured in normal gravity. Moreover, the total vessel volume was significantly higher in rats injected with CM of EPCs cultured in µ*g* compared with rats injected with CM of EPCs cultured in normal gravity. There was also a significantly higher bone volume per tissue volume in rats injected with CM of EPCs cultured in µ*g* compared with CM of EPCs cultured in normal gravity [168].

Another group [170] examined the effect of 7 days of quality and quantity culture (QQc) of human EPCs under simulated µ*g* by a 3D clinostat; the cells were cultured under normal gravity, µg, or µ*g* followed by normal conditions (ME). QQc is a way to enhance both the angiogenic potential and the number of EPCs ex vivo. There was a significant increase in the number of CD34+ cells after QQc and µ*g* and after ME compared with the control group. Moreover, there were significantly more definitive EPC colony-forming units in the ME compared with the control group, while this was not significant when the cells were only exposed to µ*g*. Similarly to Kong et al. [168], Hagiwara et al. [170] reported an upregulation of VEGF and endothelial NO synthase after QQc and µ*g* compared with the control group, but they also reported an increase in insulin-like growth factor 1, hepatocyte growth factor, fibroblast growth factor-1, matrix metalloproteinase-2, platelet-derived growth factor, leptin, and superoxide dismutase 1 compared with the control group. The ME group also showed an increase in VEGF compared with the control group.

## 6. Conclusions

Human spaceflight is associated with several cardiovascular risk factors such as changes in normal exercise routine, increased psychological stressors, and elevated exposure to ionising radiation. Upon entering u*g*, cephalad fluid shift is the most notable adjustment to space and results in an increase in SV (35–46%) and CO (18–41%). Despite these increases, astronauts enter a state of hypovolemia (10–15% decrease in blood volume) during spaceflight. The absence of orthostatic pressure and a decrease in AP reduces the workload of the heart to provide the body with oxygen and nutrient-rich blood. This is believed to be the underlying mechanism for the development of cardiac atrophy in space (10–20% decrease in ventricular size). There are also important cellular and molecular changes. Exposure to µ*g* is associated with changes in cell shape and endothelial dysfunction through suppressed cellular proliferation, increased cell apoptosis, and oxidative stress. The µ*g* platforms have allowed studying multiple physiological changes and have also been necessary for the development of appropriate tools and countermeasures for future human spaceflight missions to LEO and beyond.

## 7. Materials and Methods

For this review, a literature search was performed by using online repositories. The literature search was performed in PubMed (pubmed.ncbi.nlm.nih.gov, all accessed on 1 June 2021), Limo.libis.be, Sciencedirect.com, Frontiersin.org, Researchgate.net, Nature.com, Nasa.gov, Dlr.de, Sciencemag.org, and Clinicaltrials.gov. The search included only papers in English, Danish, and Norwegian—a factor that could lead to language bias—and because the search only included published papers, there is a risk of publication bias. Table 2 shows the number of articles found in PubMed. Additional papers were found in ‘similar articles’, ‘cited by’, and reference lists of the primary articles.

## Figures and Tables

**Table 1:** Microgravity-induced cardiovascular adaptions.

Ref	Variables	Initial Reaction	ST Response	LT Response
[2]	Blood volume	↑	↓ 10–15%	↓ 10–15%
[2,16]	Haematocrit	↑	↓ 10–15%	Unchanged or ↓
[17,18,19,20]	CO	↑	↑18–24%	↑ 41%
[19,20]	SV	↑	↑ 46%	↑ 35%
[21,22,23]	Ventricular size	↑ 20%	↓ 10%	↓ 10%
[24,25,26]	CVP	↓	↓	↓
[20,27,28,29]	MAP	Unchanged	Unchanged	↓ 10 mmHg
[20,27,28,29]	SBP	Unchanged	Unchanged	↓ 8 mmHg
[20,27,28,29]	DBP	Unchanged	↓ 5 mmHg	↓ 9 mmHg
[20,29]	SVR	Unchanged	Unchanged	↓ 39%
[24,30]	HR	Unchanged	Unchanged or↓	Unchanged or ↓
[31,32]	cIMT			↑ 10–12% (6 M)
				↑ 20% (1 Y)
[31,32]	Femoral IMT			↑ 10%–15%
[33,34,35]	Arterial stiffness			↑ 17%–30%

Abbreviations: References (Ref); short-term (ST); long-term (LT); cardiac output (CO); stroke volume (SV); central venous pressure (CVP); mean arterial pressure (MAP); systolic blood pressure (SBP); diastolic blood pressure (DBP); systemic vascular resistance (SVR); heartrate (HR); carotid intima media thickness (cIMT); femoral intima media thickness (femoral IMT); months (M); year (Y); increase (↑); decrease (↓).

**Table 2:** Number of articles found according key words (in PubMed on 3 October 2021).

Search Terms	Number of Articles
Microgravity AND Cardiovascular disease	479
Microgravity AND Radiation	1103
Radiation AND Cardiovascular disease	31,676
Radiation AND Microgravity AND Cardiovascular disease	25

## Abbreviations

ANP	Atrial natriuretic peptide
AP	Arterial pressure
AS	Atherosclerosis
APC	Atrial premature contraction
CM	Conditioned media
CO	Cardiac output
CPC	Cardiovascular progenitor cell
CVD	Cardiovascular disease
CVP	Central venous pressure
DLR	Deutsches Zentrum für Luft- und Raumfahrt
DWI	Dry water immersion
EC	Endothelial cells
ECM	Extracellular matrix
EPC	Endothelial progenitor cell
EPO	Erythropoietin
ESA	European Space Agency
FN	Fibronectin
GCR	Galactic cosmic rays
Gy	Gray
HBR	Horizontal bedrest
HDBR	Head-down bed rest
HF	High frequency
HR	Heart rate
hPSC	Human pluripotent stem cells
HRV	Heart rate variability
HUVEC	Human umbilical vein endothelial cell
IF	Intermediate filament
IL	Interleukin
IMT	Intima media thickness
ISS	International Space Station
JAMIC	Japan Microgravity Center
JAXA	Japan Aerospace Exploration Agency
LDL	Low-density lipoprotein
LEO	Low Earth orbit
LF	Low frequency
LM	Laminin
MAP	Mean arterial pressure
MCS	Multicellular spheroids
ME	Microgravity followed by normal conditions
MeV	Megaelectronvolt
mSv	Millisieverts
MSC	Mesenchymal stem cell
MT	Microtubules
NASA	National Aeronautics and Space Administration
NEEMO	NASA’s Extreme Environment Mission Operation
NGAL	Neutrophil gelatinase associated lipocalin
Ni	Nickel
NO	Nitric oxide
OI	Orthostatic intolerance
QQc	Quality and quantity culture
RANTES	Regulated on activation normal T-cell expressed and secreted
RBC	Red blood cell
RE	Earth radius
RhoA	Ras homolog family member A
RICVD	Radiation induced cardiovascular disease
ROS	Reactive oxygen species
RPM	Random positioning machine
SAA	South Atlantic Anomaly
SV	Stroke volume
Sv	Sieverts
SVR	Systemic vascular resistance
TPR	Total peripheral resistance
VDCC	Voltage-dependent Ca^2+^ channels
VEGF	Vascular endothelial growth factor
VPC	Ventricular premature contraction
VSMC	Vascular smooth muscle cell
WI	Water immersion
ZARM	Center of Applied Science and Microgravity (Bremen, Germany)
µ*g*	Microgravity
2D	Two-dimensional
3D	Three-dimensional
